# Chemical Constituents of the Ethyl Acetate Extract from *Diaphragma juglandis* Fructus and Their Inhibitory Activity on Nitric Oxide Production In Vitro

**DOI:** 10.3390/molecules23010072

**Published:** 2017-12-29

**Authors:** Dan Wang, Yan Mu, Hongjing Dong, Huijiao Yan, Cui Hao, Xiao Wang, Lisi Zhang

**Affiliations:** 1 Shandong Key Laboratory of TCM Quality Control Technology, Shandong Analysis and Test Center, Qilu University of Technology (Shandong Academy of Sciences), 19 Keyuan Street, Jinan 250014, China; wangd201610@163.com (D.W.); my3634@163.com (Y.M.); donghongjing_2006@163.com (H.D.); yanhuijiao01@163.com (H.Y.); hc56558@163.com (C.H.); 2 College of Food Science and Engineering, Shandong Agricultural University, 61 Daizong Street, Taian 271018, China; 3 Shandong Institute of Pomology, Shandong Academy of Agricultural Sciences, 66 Longtan Street, Taian 271000, China

**Keywords:** *Diaphragma juglandis* fructus, chemical constituents, high-speed counter-current chromatography, preparative separation, anti-inflammatory activity

## Abstract

*Diaphragma juglandis* fructus contains various bioactive constituents. Fourteen compounds were isolated from *Diaphragma juglandis* fructus by preparative high performance liquid chromatography (pre-HPLC) and high-speed counter-current chromatography (HSCCC). Their structures were identified by nuclear magnetic resonance (NMR) and electrospray ionization mass spectrometry (ESI-MS). Compounds (+)-dehydrovomifoliol (**12**), (6*R*,9*R*)-9-hydroxymegastigman-4-en-3-one (**13**) and (6*R*,9*S*)-9-hydroxymegastigman-4-en-3-one (**14**) are found from *Juglans regia* L. for the first time. Compounds dihydrophaseic acid (**2**), blumenol B (**3**) and (4*S*)-4-hydroxy-1-tetralone (**11**) are isolated from *Diaphragma juglandis* fructus for the first time. The anti-inflammatory effects of isolated compounds were evaluated by an in vitro model of lipopolysaccharide (LPS)-stimulated RAW 264.7 macrophages. Compounds gallic acid (**1**), ethyl gallate (**9**) and (+)-dehydrovomifoliol (**12**) exhibited inhibitory activity on the nitric oxide production of RAW 264.7 at a concentration of 25 μM. The result indicated that the combination HSCCC with pre-HPLC is an effective way for compound separation and purification. And *Diaphragma juglandis* fructus constituents have the potential for the treatment of inflammatory-related diseases.

## 1. Introduction

Walnuts (*Juglans regia* L.) are widely consumed globally due to their unique and diverse nutritional characteristics and health-related benefits such as the inhibition of arteriosclerosis, hypercholesterolaemia, cardiovascular disease [1], diabetes mellitus [2] and cancer [3]. In general, plant food processing results in accumulation of by-products that can be attractive sources for natural antioxidants and bioactive compounds. These by-products can be used in biotechnology applications to increase the value of functional ingredients [4].

*Diaphragma juglandis* fructus, which is the dry wood diaphragm of walnut kernel, is one of the well-known by-products of walnut production. *Diaphragma juglandis* is used in traditional Chinese medicine and has been used for treating several illnesses since ancient times. It is rich in a variety of bioactive components, such as phenolic acids, flavonoid, saponins, alkaloids and polysaccharides [5]. The role of the bioactive components has been widely studied in various areas. They have antioxidant [6], anti-microbial [7,8], antimutagenic [9,10], anti-inflammatory, immunomodulatory and antiplatelet effects [11]. In view of these important biological activities, a large number of compounds are urgently needed for bioactive research. Polysaccharides purified from *Diaphragma juglandis* fructus showed strong antibacterial and antioxidant activities [12]. These promising results led us to continue with further studies in order to get a deeper knowledge on *Diaphragma juglandis* fructus. However, there is insufficient information on the anti-inflammatory property of *Diaphragma juglandis* fructus.

Inflammation is an essential protective process that helps to preserve the integrity of an organism against physical, chemical and infective insults. However, excessive inflammation, including overmuch reactive nitrogen species release, can damage the normal issues or organs. Nitric oxide (NO) is one of the most important reactive nitrogen species, which is produced by inducible nitric oxide synthase in macrophages and other immune cells stimulated by lipopolysaccharide (LPS) and other stresses [13,14,15]. Thus, inhibition of the production of NO is an important target in the treatment of inflammatory diseases.

The preparative isolation of the chemical constituents of *Diaphragma juglandis* fructus extracts was established by a combination of HSCCC and pre-HPLC. The anti-inflammatory activities of isolated compounds from *Diaphragma juglandis* fructus extracts were tested in vitro using LPS-stimulated mouse RAW 264.7 macrophages.

## 2. Results and Discussion

### 2.1. Separation and Purification of Compounds

**Figure 1:**
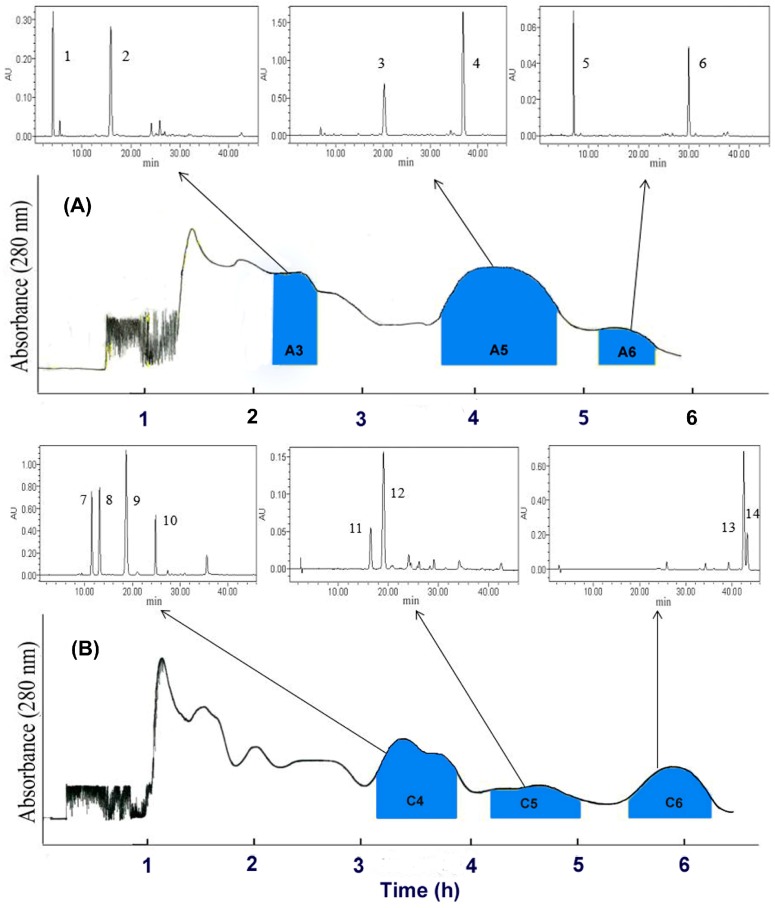
HSCCC-UV chromatograms of EtOAc extract and the upper pumped from column tail, and HPLC analysis corresponding to the HSCCC peak fractions. (**A**) Solvent systems of the first CCC run, petroleum ether-ethyl acetate-methanol-water (1:9:1:9, *v*/*v*/*v*/*v*); (**B**) Solvent systems of the second CCC run, petroleum ether-ethyl acetate-methanol-water (1:2:1:2, *v*/*v*/*v*/*v*); flow rate 2 mL/min; detection at 280 nm. A3, A5, A6, C4, C5 and C6 represent the combined major fractions.

Based on the calculated k_D_-value, petroleum ether-ethyl acetate-methanol-water (2:8:2:8, 1:9:2:8, 1:9:1:9, *v*/*v*/*v*/*v*) were tried in the first counter-current chromatography (CCC) run and the petroleum ether-ethyl acetate-methanol-water (1:3:1:3, 1:2.5:1:2.5, 1:2:1:2, *v*/*v*/*v*/*v*) were tried in the second CCC run. Finally, petroleum ether-ethyl acetate-methanol-water (1:9:1:9, *v*/*v*/*v*/*v*) was selected to separate the ethyl acetate (EtOAc) extract, and petroleum ether-ethyl acetate-methanol-water (1:2:1:2, *v*/*v*/*v*/*v*) was selected to separate the upper pumped from column tail. The efficient separation conditions of six fractions from EtOAc extract and the upper fraction pumped from the column tail were optimized and established by HSCCC. Collected fractions according to the CCC- ultraviolet (UV) chromatogram (280 nm) were analyzed by HPLC before further combination. Fractions A3 (228 mg), A5 (451 mg) and A6 (185 mg) were obtained from 3.6 g of EtOAc extract. Fractions C4 (122 mg), C5 (49 mg) and C6 (60 mg) were obtained from 1.2 g of the upper pumped from column tail. The HSCCC chromatograms of EtOAc extract and the upper pumped from column tail, and HPLC analysis of each fraction corresponding to the HSCCC peak are shown in Figure 1.

**Figure 2:**
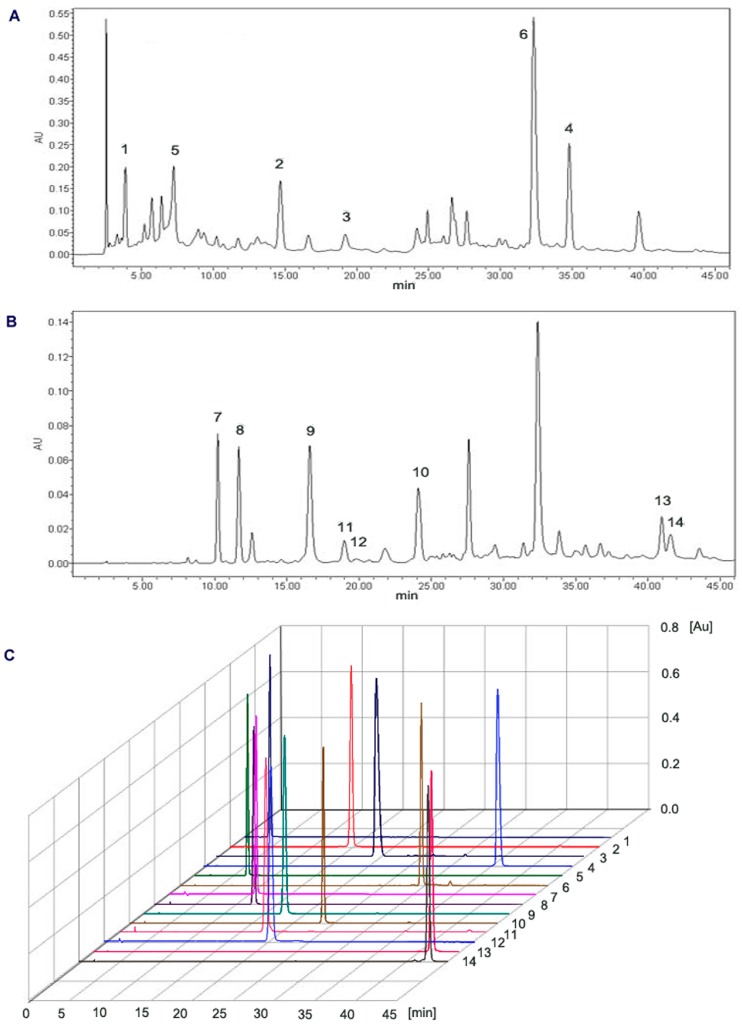
The results of HPLC analyses of EtOAc extract (**A**); the upper pumped from column tail (**B**) and purified pure compounds by pre-HPLC (**C**).

Combined fractions containing complex mixtures were further separated on the pre-HPLC after optimization of the preparative conditions. Compound **1** (12.9 mg) at 97.8% purity and **2** (26.1 mg) at 97.1% purity were obtained from 228 mg of fraction A3 after purification by pre-HPLC. Compound **3** (45.2 mg) at 97.8% purity and **4** (51.5 mg) at 98.5% purity were obtained from 451 mg of fraction A5 after purification by pre-HPLC. Compound **5** (6.5 mg) at 91.9% purity and **6** (10.8 mg) at 92.2% purity were obtained from 185 mg of fraction A6 after purification by pre-HPLC. Compound **7** (7.1 mg) at 94.1% purity, **8** (11.5 mg) at 94.4% purity, **9** (27.1 mg) at 98.0% purity and **10** (10.2 mg) at 96.3% purity were obtained from 122 mg of fraction C4 after purification by pre-HPLC. Compound **11** (1.2 mg) at 94.0% purity and **12** (5.3 mg) at 97.8% purity were obtained from 49 mg of fraction C5 after purification by pre-HPLC. Compound **13** (9.6 mg) at 95.8% purity and **14** (3.9 mg) at 92.8% purity were obtained from 60 mg of fraction C6 after purification by pre-HPLC. The results of HPLC analyses of EtOAc extract, the upper pumped from column tail and purified compounds by pre-HPLC are shown in Figure 2.

### 2.2. Structural Identification

**Figure 3:**
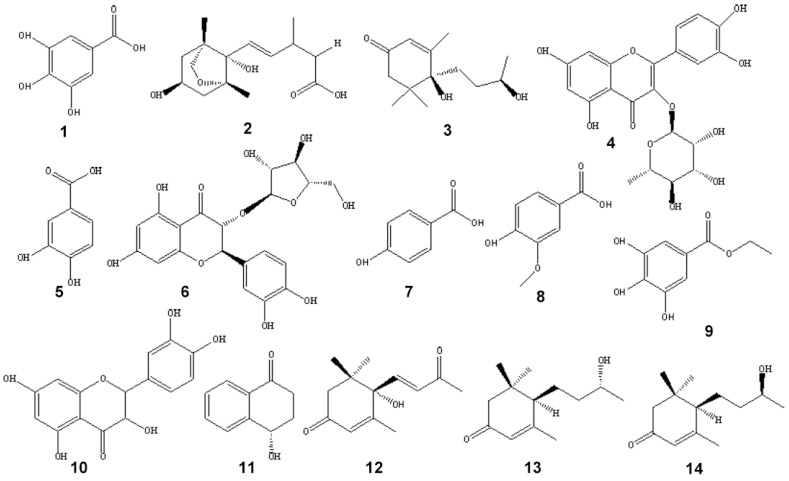
Chemical structures of compounds isolated from *Diaphragma juglandis* fructus.

Corresponding to all the isolated compounds, identification of the HPLC peak **1**–**14** were carried out by ESI-MS, ^1^H-NMR and ^13^C-NMR. The chemical structures of compounds isolated from *Diaphragma juglandis* fructus are illustrated in Figure 3.

### 2.3. The Anti-Inflammatory Activity of Isolated Compounds Based on the NO Production in LPS-Stimulated RAW 264.7 Cells

*Diaphragma juglandis* fructus contains a variety of components including phenolic acids and flavonoid as the above identified compounds. These compounds may have potent anti-inflammatory activities because *Diaphragma juglandis* fructus has been used in folk medicine to treat kidney deficiency and reproductive diseases since ancient times [12]. To confirm this hyphotesis, the anti-inflammatory activities of the isolated compounds have been evaluated herein by measuring the effect on NO production.

In this study, the initial anti-inflammatory activity screening experiment of fourteen compounds from *Diaphragma juglandis* fructus were assessed at a concentration of 50 μM. As the results show in Table 1, NO production was obviously inhibited by compounds **1** (gallic acid), **9** (ethyl gallate) and **12** ((+)-dehydrovomifoliol), which indicated potent anti-inflammatory activity (inhibition of 70% was set as a criterion). Therefore, these three compounds were selected to further investigate their effects in a dose-response experiment.

The ability of the three selected compounds at concentrations of 0.5, 2.5, 5, 25 and 50 μM to inhibit NO production in mouse RAW 264.7 macrophages was evaluated and compared with the LPS group. As shown in Table 2, LPS treatment resulted in a sharp increase in the NO level in the macrophages. Three samples were able to alleviate the LPS-stimulated NO production in a dose-dependent manner, and compound **1** showed the most NO-inhibitory effect, consistent with the initial anti-inflammatory activity screening data. These results suggest that compound **1** can be developed as an efficient anti-inflammatory agent.

## 3. Materials and Methods

### 3.1. Materials and Reagents

*Diaphragma juglandis* fructus were collected from Taishan Mountain in Shandong Province, China.

Organic solvents including methanol, ethyl acetate, petroleum ether, and ethanol were analytical grade. All solutions and dilutions water were gotten from reverse osmosis Milli-Q (Millipore, Boston, MA, USA). Methanol was chromatographic grade for HPLC analysis (Oceanpak Alexative Chemical, Co., Ltd. Göteborg, Sweden). The mouse macrophage cell line RAW 264.7 was bought from American Type Culture Collection (Virgina, VA, USA). Griess Reagent System was purchased from Beyotime Biotechnology in Beijing, China.

### 3.2. Apparatus

HSCCC was installed by TBE 300C (Tauto Biotech, Shanghai, China) and a multilayer I.D. 1.6 mm coil for a total capacity of 300 mL. The rotation speed could be controlled from 0 to 1000 rpm. TBP5002 pump (Tauto Biotech, Shanghai, China) pumped the solvent into the column. Continuous monitoring of the eluent was detected with 8823B-UV Monitor (Beijing BINTA Instrument Technology, Beijing, China) at 280 nm. A manual sample injection valve installed 20 mL loop (Tauto Biotech, Shanghai, China) was used to inject sample into the column. A recording instrument (Yokogawa Model 3057, Chongqing, China) was used for mapping.

Samples were analyzed on the Waters Alliance 2695 series made in USA, including a four binary gradient pump, 2998 Photodiode Array Detector (PAD), 2695 system controller, and an empower 3 work-station (Waters, Milford, MA, USA).

The nuclear magnetic resonance (NMR) spectra was obtained by Bruker-400 MHz NMR (Madison, WI, USA) and Varian INOVA-600 MHz NMR (Palo Alto, CA, USA), and spectrometer with TMS as the internal standard. Electrospray ionization mass spectrometry (ESI-MS) (Madison, WI, USA) was obtained from an Agilent 1100/MSG1946 mass spectrometer (Palo Alto, CA, USA).

### 3.3. Preparation of the Crude Extracts

*Diaphragma juglandis* fructus (9 kg) were milled to powder by a grinder. The powdered material was extracted with 95% ethanol under reflux for 2 h, repeated two times. Then the extract was filtered using a vacuum filtration device. The combined aqueous ethanol extract was evaporated using a rotary evaporator, to an aqueous fraction, diluted with 2 L water. Then, it was extracted with an equal volume of petroleum ether, and this was repeated six times. The aqueous layer was subsequently extracted with an equal volume of ethyl acetate, repeated eight times. Solvent evaporated and the residue was freeze-dried to afford 47.3 g of EtOAc extract.

### 3.4. Separation and Purification of the Ethyl Acetate Extract

#### 3.4.1. Solvent Selection and HSCCC Separation

In the chromatographic separation process of HSCCC, the most important step was the choice of the most efficient two-phase solvent system for the target compound(s) [16]. The choice of solvent system is based on the partition of a solute between the two kinds of liquids including the mobile and stationary phases. A series of solvent systems were tested by HPLC in order to achieve effective separation of the target components based on the tested K_D_-values.

The selected solvent system was repeated vigorous shaking in a separation funnel at room temperature then waiting for thoroughly equilibrated. Two phases were separated shortly and each phase was degassed by ultrasound for 10 min. The heavier denser phase was used for the mobile phase, while the lighter phase was used as the stationary phase. The crude extract was dissolved in a solution composed of the upper and lower phases (1:1, *v*/*v*) for the HSCCC separation.

The stationary phase pumped into the entire column at 20 mL/min, and then pumped the mobile phase into the column at 2 mL/min when the device was rotated at 800 rpm. When two phases reached the hydrodynamic equilibrium, a clear mobile phase emerged at the tail; the sample solution (200–600 mg in 10 mL of a mixture 1:1 of upper and lower phases) was injected through the injection valve. Then UV detection was performed at 280 nm from the outlet of the column and each peak fraction was collected in 10 mL glass tubes according to the peak in the chromatogram. When the separation was finished, the stationary phase in the column was rushed out by pressurized nitrogen and gleaned in a graduated cylinder to gauge the retention volume.

#### 3.4.2. Fractionation of the Ethyl Acetate Extract

The first CCC (CCC 1) of the EtOAc extract (600 mg/batch) was conducted by petroleum ether-ethyl acetate-methanol-water (1:9:1:9, *v*/*v*/*v*/*v*), collecting 45 × 10 mL fractions over 6 h according to the UV chromatogram recorded at 280 nm in the CCC monitor. Samples were subjected to HPLC and those with similar profiles were combined to afford three main fractions (A3, A5 and A6). In order to scale up the isolation, the procedure was repeated several times which can provide adequate samples for subsequent separation.

The upper pumped from column tail had complex fractions as indicated by HPLC, were subjected to a second CCC (CCC 2) after a solvent selection procedure. It was fractionated on the CCC (400 mg/batch) by petroleum ether-ethyl acetate-methanol-water (1:2:1:2, *v*/*v*/*v*/*v*), collecting 50 × 10 mL fractions over 6.5 h according to the UV chromatogram recorded at 280 nm in the CCC monitor. Three main fractions (C4, C5 and C6) were obtained after combining similar fractions based on HPLC analysis. An overview of the separation process is summarized in Figure 1.

#### 3.4.3. Purification by Pre-HPLC

**Figure 4:**
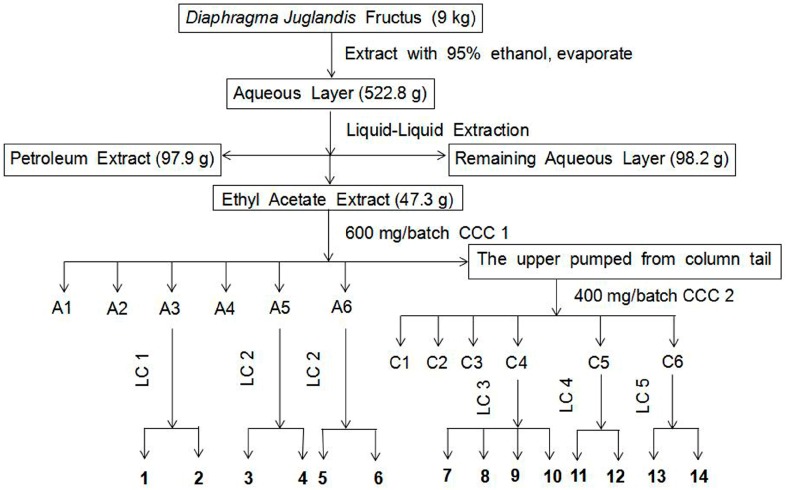
Overall work flow for isolation using HSCCC and purification by pre-HPLC of compounds from *Diaphragma juglandis* fructus. CCC 1: petroleum ether-ethyl acetate-methanol-water (1:9:1:9, *v*/*v*/*v*/*v*); CCC 2: petroleum ether-ethyl acetate-methanol-water (1:2:1:2, *v*/*v*/*v*/*v*); LC 1: 30% methanol (A)–70% 0.1% formic acid in H_2_O (B), LC 2: 40% A–60% B, LC 3: 32% A–68% B, LC 4: 35% A–65% B, LC 5: 50% A–50% B.

The pre-HPLC separation was performed using a C_18_ reverse-phase column (YMC-PEAK ODS-A column, 250 mm × 10.0 mm, 5 μm) with a binary solvent system comprising of methanol (A) and 0.1% formic acid in H_2_O (B). The flow rate was 3 mL/min. Purification of each fraction from HSCCC was conducted using an isocratic elution with different rate A and B: A3 was performed LC 1 (30% A–70% B), A5 and A6 were performed LC 2 (40% A–60% B), C4 was performed LC 3 (32% A–68% B), C5 was performed LC 4 (35% A–65% B), C6 and C7 were performed LC 5 (50% A–50% B). If necessary, it was re-purified by pre-HPLC. The flow chart of extraction, separation and purification is detailed in Figure 4.

### 3.5. Analysis and Identification of Isolated Pure Compounds

The sample which was pre-purified, fractions isolated from the HSCCC and purified pure compounds by pre-HPLC were analyzed by HPLC on a YMC-PEAK ODS-A column (250 mm × 4.6 mm, 5 μm) at the column temperature 25 °C. Methanol (solvent A) and 0.1% formic acid in H_2_O (solvent B) were served as the mobile phase. The flow rate was set at 1 mL/min and the step gradient was set as 25–30% A for 5 min, isocratic at 30% A over 15 min, and return to 40% A over 1 min, 40–50% A for 19 min, isocratic at 50% A over 5 min. The eluent was detected by a PAD at 280 nm.

The isolated compounds were identified based on a combination of nuclear magnetic resonance (NMR) analyses, electrospray ionization mass spectrometry (ESI-MS), and with the aid of published data and available reference standards. The detail data of each compound was as the following:

Compound **1** (peak **1** in Figure 2A): ESI-MS (*m*/*z*): 169 [M − H]^−^, 171 [M + H]^+^, molecular formula C_7_H_6_O_5_, M = 170; ^1^H-NMR (CD_3_OD-*d*_6_, 400 Hz) δ: 7.06 (2H, s, H-2, 6), 4.92 (-OH); ^13^C-NMR (CD_3_OD-*d*_6_, 100 Hz) δ: 169.0 (C-7), 145.0 (C-3, 5), 138.2 (C-4), 120.6 (C-1), 108.9 (C-2, 6). Comparing the above data with the literature [17,18], compound **1** was identified as gallic acid.

Compound **2** (peak **2** in Figure 2A): Negative ESI-MS (*m*/*z*): 281 [M − H]^−^, molecular formula C_15_H_22_O_5_, M = 282; ^1^H-NMR (CD_3_OD-*d*_6_, 400 MHz) δ: 7.96 (1H, d, *J* = 16.0 Hz, H-4), 6.50 (1H, d, *J* = 15.6 Hz, H-5), 5.77 (3H, s, H-2), 3.81 (1H, dd, *J* = 2.0, 2.0 Hz, H-9), 3.71 (1H, d, *J* = 7.6 Hz, H-12a), 3.31 (1H, m, H-12b), 1.85 (1H, dd, *J* = 7.2, 13.6 Hz, H-8a), 1.73 (1H, dd, *J* = 10.4, 10.4 Hz, H-8b), 1.66 (1H, m, H-10a), 1.62 (1H, d, *J* = 2.0 Hz, H-10b), 2.07 (3H, s, H-15), 0.93 (3H, s, H-14), 1.15 (3H, s, H-13); ^13^C-NMR (CD_3_OD-*d*_6_, 100 MHz) δ: 168.7 (C-1), 149.3 (C-3), 133.4 (C-5), 130.5 (C-4), 118.5 (C-2), 86.4 (C-7), 81.8 (C-6), 75.9 (C-12), 64.6 (C-9), 48.3 (C-11), 44.6 (C-8), 43.2 (C-10), 19.8 (C-15), 18.3 (C-14), 15.0 (C-13). Comparing the above data with the literature [19], compound **2** was identified as dihydrophaseic acid.

Compound **3** (peak **3** in Figure 2A): ESI-MS (*m*/*z*): 249 [M + Na]^+^, 190 [M − Cl]^−^, molecular formula C_13_H_22_O_3_, M = 226; ^1^H-NMR (CD_3_OD-*d*_6_, 400 MHz) δ: 1.03 (3H, d, *J* = 7.6 Hz, H-12), 1.13 (3H, m, H-13), 1.24 (3H, m, H-10), 1.42 (1H, m, H-8b), 1.66 (1H, m, H-8a), 1.77 (1H, m, H-7a), 1.94 (1H, m, H-7b), 2.03 (3H, d, *J* = 1.6 Hz, H-11), 2.16 (1H, d, *J* = 18.0 Hz, H-2b), 2.58 (1H, d, *J* = 18.0 Hz, H-2a), 3.65 (1H, m, H-9), 5.83 (1H, m, H-4); ^13^C-NMR (CD_3_OD-*d*_6_, 100 MHz) δ: 199.5 (C-3), 170.3 (C-5), 125.2 (C-4), 77.7 (C-6), 67.5 (C-9), 49.7 (C-2), 41.5 (C-1), 33.9 (C-7), 33.8 (C-8), 23.2 (C-13), 22.6 (C-12), 22.1 (C-10), 20.4 (C-11). Comparing the above data with the literature [20], compound **3** was identified as blumenol B.

Compound **4** (peak **4** in Figure 2A): ESI-MS (*m*/*z*): 447 [M − H]^−^, 449 [M + H]^+^, 471 [M + Na]^+^, molecular formula C_21_H_20_O_11_, M = 448; ^1^H-NMR (CD_3_OD-*d*_6_, 400 MHz) δ: 7.34 (1H, d, *J* = 2.4 Hz, H-2), 7.31 (1H, dd, *J* = 2.4, 2.0 Hz, H-6), 6.91 (1H, d, *J* = 8.4 Hz, H-5), 6.37 (1H, d, *J* = 2.4 Hz, H-8), 6.20 (1H, d, *J* = 2.0 Hz, H-6), 5.35 (1H, d, *J* = 1.2 Hz, H-1), 4.22 (1H, dd, *J* = 1.6, 1.6 Hz, H-2), 3.74 (1H, dd, *J* = 3.2, 3.6 Hz, H-3), 3.31 (1H, t, *J* = 9.0 Hz, H-4), 3.42 (1H, m, H-5), 0.94 (3H, d, *J* = 6.0 Hz, H-6); ^13^C-NMR (CD_3_OD-*d*_6_, 100 MHz) δ: 178.2 (C-4), 164.5 (C-7), 161.8 (C-5), 157.9 (C-9), 157.1 (C-2), 148.4 (C-4′), 145.0 (C-3′), 134.8 (C-3), 121.6 (C-6′), 121.5 (C-1′), 115.5 (C-5′), 115.0 (C-2′), 104.5 (C-10), 102.1 (C-1″), 98.4 (C-6), 93.3 (C-8), 71.8 (C-4″), 70.7 (C-3″), 70.6 (C-2″), 70.5 (C-5″), 16.2 (C-6″). Comparing the above data with the literature [21], compound **4** was identified as quercitrin.

Compound **5** (peak **5** in Figure 2A): ESI-MS (*m*/*z*): 155 [M + H]^+^, 153 [M − H]^−^, molecular formula C_7_H_6_O_4_, M = 154; ^1^H-NMR (CD_3_OD-*d*_6_, 400 MHz) δ: 6.77 (1H, d, *J* = 8.0 Hz, H-5), 7.40 (1H, m, H-2), 7.43 (1H, d, *J* = 1.6 Hz, H-6); ^13^C-NMR (CD_3_OD-*d*_6_, 100 MHz) δ: 170.2 (-COOH), 149.5 (C-4), 144.5 (C-3), 123.6 (C-1), 122.3 (C-6), 116.4 (C-2), 114.2 (C-5). Comparing the above data with the literature [22,23], compound **5** was identified as protocatechuic acid.

Compound **6** (peak **6** in Figure 2A): ESI-MS (*m*/*z*): 459 [M + Na]^+^, 435 [M − H]^−^, 471 [M − Cl]^−^; ^1^H-NMR (CD_3_OD-*d*_6_, 400 MHz) δ: 7.00 (1H, t, H-2′), 6.88 (1H, dd, *J* = 1.6, 2.0 Hz, H-6′), 6.82 (1H, d, *J* = 8.0 Hz, H-5′), 5.93 (1H, d, *J* = 2.0 Hz, H-8), 5.90 (1H, m, H-6), 5.05 (1H, d, *J* = 3.6 Hz, H-2), 5.02 (1H, d, *J* = 3.6 Hz, H-3), 4.20 (1H, d, *J* = 4.4 Hz, H-1″), 3.91 (1H, s, H-2″), 3.78 (1H, s, H-3″), 4.24 (1H, m, H-4″), 3.60 (1H, m, H-5″); ^13^C-NMR (CD_3_OD-*d*_6_, 100 MHz) δ: 196.1 (C-4), 167.8 (C-7), 164.0 (C-5), 163.2 (C-9), 146.2 (C-4′), 145.2 (C-3′), 127.8 (C-1′), 119.4 (C-6′), 114.9 (C-5′), 114.3 (C-2′), 100.8 (C-10), 96.1 (C-6), 95.1 (C-8), 82.5 (C-2), 73.4 (C-3), 106.4 (C-1″), 79.8 (C-2″), 77.9 (C-3″), 87.5 (C-4″), 61.9 (C-5″). Comparing the above data with the literature [24,25], compound **6** was identified as taxifolin-3-*O*-α-l-arabinofuranoside.

Compound **7** (peak **7** in Figure 2B): ESI-MS (*m*/*z*): 139 [M + H]^+^, 137 [M − H]^−^, molecular formula C_7_H_6_O_3_, M = 138; ^1^H-NMR (CD_3_OD-*d*_6_, 400 MHz) δ: 7.87 (2H, d, *J* = 8.8 Hz, H-3, 5), 6.81 (2H, d, *J* = 8.8 Hz, H-2, 6); ^13^C-NMR (CD_3_OD-*d*_6_, 100 MHz) δ: 121.5 (C-1), 114.6 (C-2, 6), 131.6 (C-3, 5), 162.0 (C-4), 168.8 (C-7). Comparing the above data with the literature [26,27], compound **7** was identified as *p*-hydroxybenzoic acid.

Compound **8** (peak **8** in Figure 2B): ESI-MS (*m*/*z*): 169 [M + H]^+^, 167 [M − H]^−^, molecular formula C_8_H_8_O_4_, M = 168; ^1^H-NMR (CD_3_OD-*d*_6_, 400 MHz) δ: 7.56 (1H, d, *J* = 2.0 Hz, H-6), 7.55 (1H, d, *J* = 2.8 Hz, H-2), 6.83 (1H, d, *J* = 8.8 Hz, H-5), 3.89 (3H, s, -OCH_3_); ^13^C-NMR (CD_3_OD-*d*_6_, 100 MHz) δ: 168.7 (C-7), 151.3 (C-4), 147.3 (C-3), 123.9 (C-6), 121.8 (C-1), 114.4 (C-2), 112.4 (C-5), 55.0 (-OCH_3_). Comparing the above data with the literature [28,29], compound **8** was identified as vanillic acid.

Compound **9** (peak **9** in Figure 2B): ESI-MS (*m*/*z*): 199 [M + H]^+^, 197 [M − H]^−^, molecular formula C_9_H_10_O_5_, M = 198; ^1^H-NMR (CD_3_OD-*d*_6_, 400 MHz) δ: 7.04 (2H, s, H-2, 6), 4.27 (2H, q, -OCH_2_-), 1.34 (3H, t, -CH_3_); ^13^C-NMR (CD_3_OD-*d*_6_, 100 MHz) δ: 13.2 (C-9), 60.3 (C-8), 108.7 (C-2, 6), 120.4 (C-1), 138.3 (C-4), 145.1 (C-3, 5), 167.2 (C-7). Comparing the above data with the literature [30], compound **9** was identified as ethyl gallate.

Compound **10** (peak **10** in Figure 2B): ESI-MS (*m*/*z*): 305 [M + H]^+^, 327 [M + Na]^+^, 303 [M − H]^−^, molecular formula C_15_H_12_O_7_, M = 304; 1H-NMR (CD_3_OD-*d*_6_, 400 MHz) δ: 4.88 (1H, d, *J* = 10.0 Hz, H-2), 6.96 (1H, d, *J* = 2.0 Hz, H-2′), 4.48 (1H, s H-3), 6.80 (1H, d, *J* = 8.0 Hz, H-5′), 5.88 (1H, m, H-6), 6.84 (1H, dd, *J* = 2.0, 2.0 Hz, H-6′), 5.92 (1H, d, *J* = 2.0 Hz, H-8); ^13^C-NMR (CD_3_OD-*d*_6_, 100 MHz) δ: 197.0 (C-4), 167.4 (C-7), 163.9 (C-5), 163.1 (C-9), 145.7 (C-4′), 144.9 (C-3′), 128.5 (C-1′), 119.5 (C-6′), 114.7 (C-5′), 114.5 (C-2′), 100.4 (C-10), 95.9 (C-6), 94.9 (C-8), 83.7 (C-2), 72.3 (C-3). Comparing the above data with the literature [31], compound **10** was identified as dihydroquercetin.

Compound **11** (peak **11** in Figure 2B): ESI-MS (*m*/*z*): 163 [M + H]^+^, molecular formula C_10_H_10_O_2_, M = 162; ^1^H-NMR (CD_3_OD-*d*_6_, 600 MHz) δ: 2.65 (1H, ddd, *J* = 5.4, 2.4, 5.4 Hz, H-2), 2.88 (1H, ddd, *J* = 3.0, 6.0, 3.0 Hz, H-2), 2.15 (1H, m, H-3), 2.38 (1H, m, H-3), 4.94 (1H, dd, *J* = 4.2, 4.2 Hz, H-4), 7.65 (2H, m, H-5, 6), 7.44 (1H, m, H-7), 7.97 (1H, d, *J* = 7.8 Hz, H-8); ^13^C-NMR (CD_3_OD-*d*_6_, 150 MHz) δ: 198.5 (C-1), 146.5 (C-10), 133.8 (C-6), 130.9 (C-9), 127.6 (C-8), 127.1 (C-7), 126.2 (C-5), 66.9 (C-4), 34.9 (C-2), 31.7 (C-3). Comparing the above data with the literature [32], compound **11** was identified as (4*S*)-4-hydroxy-1-tetralone.

Compound **12** (peak **12** in Figure 2B): ESI-MS (*m*/*z*): 245 [M + Na]^+^, 223 [M + H]^+^; molecular formula C_13_H_18_O_3_, M = 222; ^1^H-NMR (CD_3_OD-*d*_6_, 600 MHz) δ: 2.32 (3H, s, 10-CH_3_), 1.08 (3H, s, 11-CH_3_), 1.04 (3H, s, 12-CH_3_), 1.92 (3H, d, *J* = 1.2 Hz, 13-CH_3_), 2.29 (1H, d, *J* = 16.8 Hz, 2b-H), 2.62 (1H, d, *J* = 17.4 Hz, 2a-H), 5.95 (1H, m, 4-H), 6.45 (1H, d, *J* = 16.2 Hz, 8-H), 7.01 (1H, d, *J* = 15.6 Hz, 7-H); ^13^C-NMR (CD_3_OD-*d*_6_, 150 MHz) δ: 41.2 (C-1), 49.1 (C-2), 198.9 (C-3), 126.6 (C-4), 163.3 (C-5), 78.6 (C-6), 146.9 (C-7), 130.3 (C-8), 199.3 (C-9), 26.3 (C-10), 22.1 (C-11), 23.4 (C-12), 17.8 (C-13). Comparing the above data with the literature [33], compound **12** was identified as (+)-dehydrovomifoliol.

Compound **13** (peak **13** in Figure 2B): ESI-MS (*m*/*z*): 233 [M + Na]^+^, 211 [M + H]^+^; ^1^H-NMR (CD_3_OD-*d*_6_, 600 MHz) δ: 5.83 (1H, s, H-1), 2.47 (1H, d, *J* = 17.4 Hz, H-4a), 2.02 (1H, d, *J* = 14.4 Hz, H-4b), 1.99 (1H, m, H-6), 1.94 (1H, m, H-7a), 1.46 (1H, m, H-7b), 1.55 (2H, m, H-8), 3.71 (1H, dd, *J* = 6.6, 6.0 Hz, H-9), 1.19 (3H, d, *J* = 6.20 Hz, H-10), 1.11 (3H, s, H-11), 1.04 (3H, s, H-12), 2.06 (3H, d, *J* = 1.2 Hz, H-13); ^13^C-NMR (CD_3_OD-*d*_6_, 150 MHz) δ: 200.8 (C-3), 168.3 (C-5), 124.0 (C-4), 67.1 (C-2), 51.0 (C-6), 46.7 (C-2), 38.4 (C-8), 35.9 (C-1), 27.6 (C-12), 26.1 (C-11), 25.9 (C-7), 23.5 (C-13), 22.2 (C-10). Comparing the above data with the literature [34,35], compound **13** was identified as (6*R*, 9*R*)-9-hydroxymegastigman-4-en-3-one.

Compound **14** (peak **14** in Figure 2B): ESI-MS (*m*/*z*): 233 [M + Na]^+^, 211 [M + H]^+^; ^1^H-NMR (CD_3_OD-*d*_6_, 600 MHz) δ: 5.83 (1H, s, H-1), 2.47 (1H, d, *J* = 17.4 Hz, H-4a), 2.02 (1H, d, *J* = 4.8 Hz, H-4b), 2.00 (1H, m, H-6), 1.78 (1H, m, H-7a), 1.63 (1H, m, H-7b), 1.55 (2H, m, H-8), 3.71 (1H, dd, *J* = 6.0, 6.0 Hz, H-9), 1.18 (3H, d, *J* = 6.6 Hz, H-10), 1.11 (3H, s, H-11), 1.04 (3H, s, H-12), 2.06 (3H, d, *J* = 1.8 Hz, H-13); ^13^C-NMR (CD_3_OD-*d*_6_, 150 MHz) δ: 200.8 (C-3), 168.3 (C-5), 124.0 (C-4), 67.4 (C-2), 51.1 (C-6), 46.7 (C-2), 38.4 (C-8), 35.9 (C-1), 27.6 (C-12), 26.1 (C-11), 26.0 (C-7), 23.5 (C-13), 22.1 (C-10). Comparing the above data with the literature [34,35], compound 14 was identified as (6*R*, 9*S*)-9-hydroxymegastigman-4-en-3-one.

### 3.6. Anti-Inflammatory Activity Assay against NO Production

The inhibitory activity against NO production was assayed mainly using the method described in the literature by Zhou [36]. The mouse macrophage cell line RAW 264.7 was cultured in DMEM supplemented with 10% FBS, 100 μg/mL streptomycin and 100 U/mL penicillin in a humidified incubator with 5% CO_2_ at 37 °C.

The initial anti-inflammatory activity screening experiment of fourteen compounds from *Diaphragma juglandis* fructus were assessed at 50 μM concentration. Samples were dissolved in DMSO before addition to cell cultures, and the final ratio of DMSO to medium was 0.1% (*v*/*v*). Cells (1.5 × 10^4^ cells in 100 μL DMEM per well) were seeded onto 96-well plates and cultured for 24 h. Then DMEM was removed and replaced with fresh medium containing 1 μg/mL [36,37] of LPS (DMSO was used as blank control) and 50 μM of different compounds. After 24 h incubation, the cell-free culture medium was collected, mixed with an equal volume of Griess reagent (1% sulfanilamide and 0.1% *N*-1-naphthylenediamine dihydrochloride in 2.5% phosphoric acid) for NO determination using the Griess reaction. The absorbance value (OD) at 540 nm was read on a microplate reader by a colorimetric assay. The compounds that exhibited anti-inflammatory activity at 50 μM were selected to further investigate in the dose-response experiment (0.5, 2.5, 5, 25 and 50 μM concentrations) according to the above procedure. The NO production was measured by a standard curve generated using serial dilutions of NaNO_2_ in fresh culture medium (R^2^ = 0.999). The results are expressed as the mean ± SD of three independent experiments.

Statistical analysis was performed using Microsoft Office Excel 2010. Differences between the two groups were analyzed using the two-tails Student’s *t*-test. Differences were considered significant at a probability level of 95% or greater (*p* ≤ 0.05). In tables, all values are expressed as mean ± SD.

## 4. Conclusions

The study presented in this paper indicated that *Diaphragma juglandis* fructus contains various bioactive constituents. Compounds that have been isolated were gallic acid (**1**), dihydrophaseic acid (**2**), blumenol B (**3**), quercitrin (**4**), protocatechuic acid (**5**), taxifolin-3-*O*-α-l-arabinofuranoside (**6**), *p*-hydroxybenzoic acid (**7**), vanillic acid (**8**), ethyl gallate (**9**), dihydroquercetin (**10**), (4*S*)-4-hydroxy-1-tetralone (**11**), (+)-dehydrovomifoliol (**12**), (6*R*,9*R*)-9-hydroxymegastigman-4-en-3-one (**13**), (6*R*,9*S*)-9-hydroxymegastigman-4-en-3-one (**14**). Compounds **12**, **13** and **14** are described in *Juglans regia* L. for the first time. Compounds **2**, **3** and **11** are isolated from *Diaphragma juglandis* fructus for the first time. Combination of HSCCC with pre-HPLC is an effective method that would be applicable to the separation and purification of compounds.

Nitric oxide radical inhibition assay suggested that gallic acid, ethyl gallate and (+)-dehydrovomifoliol are major components of *Diaphragma juglandis* fructus that exert anti-inflammatory effect. These results revealed that *Diaphragma juglandis* fructus possesses potential for the treatment of inflammatory-related diseases and application developments.

Thus, using *Diaphragma juglandis* fructus as a source of bioactive components for medical purposes will increase the value of walnut production, as well as offer utilization for a by-product, which is produced in a large quantity.

## Figures and Tables

**Table 1:** The NO production relative to LPS group of isolated compounds in LPS-stimulated RAW 264.7 macrophages.

Group		NO Production Relative to LPS Group (Mean ± SD)
Control (DMSO)		0.23 ± 0.03
LPS (1 μg/mL)		1 ± 0
Compounds (50 μM)	**1**	0.59 ± 0.05
	**2**	0.97 ± 0.07
	**3**	0.93 ± 0.07
	**4**	0.95 ± 0.06
	**5**	0.63 ± 0.09
	**6**	0.99 ± 0.08
	**7**	0.61 ± 0.1
	**8**	0.81 ± 0.04
	**9**	0.57 ± 0.04
	**10**	0.92 ± 0.03
	**11**	0.72 ± 0.03
	**12**	0.62 ± 0.05
	**13**	0.98 ± 0.05
	**14**	0.86 ± 0.07

**Table 2:** The NO production of isolated compounds at different concentrations in LPS-stimulated RAW 264.7 macrophages.

Group			NO Concentration (μM) (Mean ± SD)
Control (DMSO)			3.13 ± 0.67
LPS (1 μg/mL)			38.47 ± 0.82
Compounds	**1**	0.5 μM	28.05 ± 1.87
		2.5 μM	27.78 ± 1.79
		5 μM	26.28 ± 1.72
		25 μM	25.20 ± 1.41
		50 μM	21.45 ± 1.71
	**9**	0.5 μM	40.16 ± 2.05
		2.5 μM	35.60 ± 1.73
		5 μM	28.25 ± 2.22
		25 μM	23.23 ± 1.93
		50 μM	22.3 ± 1.44
	**12**	0.5 μM	37.01 ± 1.34
		2.5 μM	33.69 ± 1.86
		5 μM	32.31 ± 2.01
		25 μM	26.47 ± 2.10
		50 μM	22.97 ± 1.95
